# Navigating Diagnostic and Treatment Decisions in Non‐Small Cell Lung Cancer: Expert Commentary on the Multidisciplinary Team Approach

**DOI:** 10.1002/onco.13586

**Published:** 2020-11-21

**Authors:** Sanjay Popat, Neal Navani, Keith M. Kerr, Egbert F. Smit, Timothy J.P. Batchelor, Paul Van Schil, Suresh Senan, Fiona McDonald

**Affiliations:** ^1^ Lung Unit, Royal Marsden Hospital London United Kingdom; ^2^ The Institute of Cancer Research, University of London London United Kingdom; ^3^ Lungs for Living Research Centre, University College London (UCL) Respiratory, UCL and Department of Thoracic Medicine, University College London Hospitals NHS Foundation Trust London United Kingdom; ^4^ Department of Pathology, Aberdeen University Medical School and Aberdeen Royal Infirmary Aberdeen United Kingdom; ^5^ Department of Pulmonary Diseases, VU University Medical Center and Department of Thoracic Oncology, The Netherlands Cancer Institute Amsterdam The Netherlands; ^6^ Department of Thoracic Surgery, University Hospitals Bristol and Weston National Health Service Foundation Trust Bristol United Kingdom; ^7^ Department of Thoracic and Vascular Surgery, Antwerp University Hospital and Antwerp University Antwerp Belgium; ^8^ Department of Radiation Oncology, Amsterdam University Medical Center, Free University Amsterdam, Cancer Center Amsterdam Amsterdam The Netherlands

**Keywords:** Non‐small cell lung cancer, Multidisciplinary, Decision making, Lung cancer, Treatment guidelines

## Abstract

Non‐small cell lung cancer (NSCLC) accounts for approximately one in five cancer‐related deaths, and management requires increasingly complex decision making by health care professionals. Many centers have therefore adopted a multidisciplinary approach to patient care, using the expertise of various specialists to provide the best evidence‐based, personalized treatment. However, increasingly complex disease staging, as well as expanded biomarker testing and multimodality management algorithms with novel therapeutics, have driven the need for multifaceted, collaborative decision making to optimally guide the overall treatment process. To keep up with the rapidly evolving treatment landscape, national‐level guidelines have been introduced to standardize patient pathways and ensure prompt diagnosis and treatment. Such strategies depend on efficient and effective communication between relevant multidisciplinary team members and have both improved adherence to treatment guidelines and extended patient survival. This article highlights the value of a multidisciplinary approach to diagnosis and staging, treatment decision making, and adverse event management in NSCLC.

**Implications for Practice:**

This review highlights the value of a multidisciplinary approach to the diagnosis and staging of non‐small cell lung cancer (NSCLC) and makes practical suggestions as to how multidisciplinary teams (MDTs) can be best deployed at individual stages of the disease to improve patient outcomes and effectively manage common adverse events. The authors discuss how a collaborative approach, appropriately leveraging the diverse expertise of NSCLC MDT members (including specialist radiation and medical oncologists, chest physicians, pathologists, pulmonologists, surgeons, and nursing staff) can continue to ensure optimal per‐patient decision making as treatment options become ever more specialized in the era of biomarker‐driven therapeutic strategies.

## Abstract 2

This article highlights the value of a multidisciplinary approach to diagnosis and staging, treatment decision making, and adverse event management in non‐small cell lung cancer.

## Introduction

Lung cancer represents a major public health burden. A majority of cases present as advanced non‐small cell lung cancer (NSCLC) [1, 2], with wide variability in biology, and tumor burden at presentation, and in prognosis for any given pathology and stage [3, 4]. Moreover, despite appropriate staging, many patients may be unsuitable for optimal treatments, necessitating per‐patient–level discussions about suitable treatment modalities to ensure optimal outcomes.

The NSCLC treatment landscape has been evolving rapidly over the past decade, with targeted therapies [5] and immune checkpoint inhibitors (alongside advanced radiation techniques) joining traditional chemotherapy (CTx), radiation therapy (RTx), and surgery as key components of disease management. Treatment recommendations are therefore highly dependent on patients’ tumor and biomarker characteristics, alongside stage and physiology [3, 6, 7]. Therefore, a sound understanding of current therapy data is important and can be used alongside all relevant patient data and local and regional guidelines to make the appropriate diagnostic and therapeutic decisions throughout treatment, especially where scientific data may be lacking.

**Figure 1:**
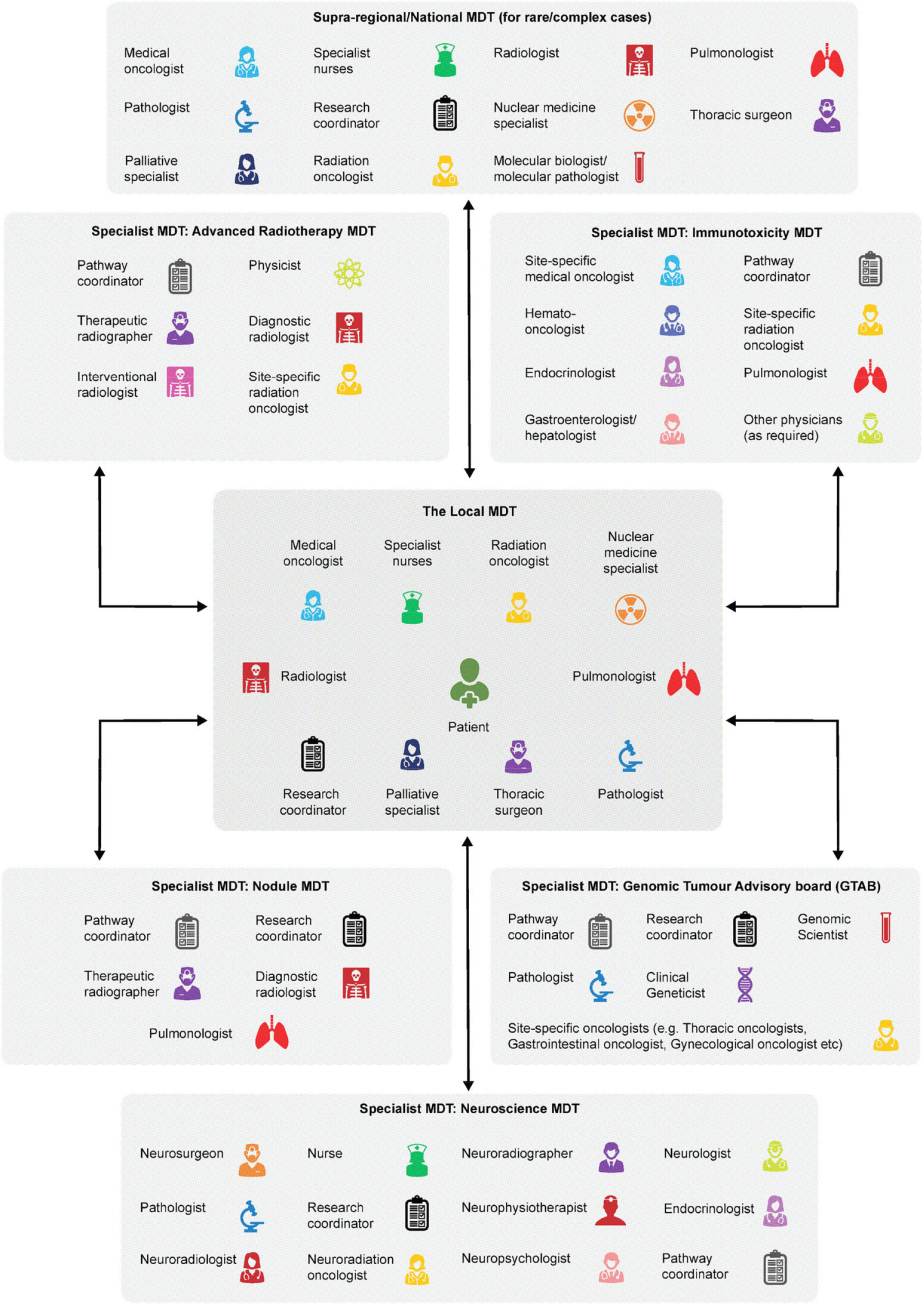
Key members of the lung cancer MDT, highlighting specialist and supra‐regional/national MDTs contributing to patient care and their potential composition. Abbreviation: MDT, multidisciplinary team.

Although many treatment recommendations can be protocolized, multifaceted treatment options have driven the need for a multidisciplinary team (MDT) to manage the full spectrum of the patient treatment journey, especially for the increasing burden of complex cases that may not neatly fit into standard diagnostic/treatment algorithms. Moreover, MDT working is increasingly recognized as a standard for high‐quality cancer care [8]. Lung cancer‐specific MDTs therefore require insight from a diverse group of specialists, including pulmonologists, thoracic surgeons, radiologists, medical oncologists, pathologists, radiation oncologists, nuclear medicine specialists, palliative care specialists, and specialist nurses (Fig. 1) [9, 10, 11]. In specific instances, this core group will also require the expertise of other specialists, including immunologists, dermatologists, gastroenterologists, endocrinologists, neurologists, cardiologists, nephrologists, and molecular biologists [12].

The need for a multidisciplinary approach is particularly evident for patients with stage III NSCLC. Some studies suggest that the benefits of MDTs are most notable in patients with more advanced (stage III or IV) NSCLC, but we argue for MDT involvement for patients with early‐stage tumors as well [2, 13], for example in maximizing radical treatment options among patients with borderline operable disease due to comorbidities, or in those with multifocal lung changes [14, 15]. Indeed, survival rates of patients with NSCLC are positively correlated with use of active treatment [16], and deployment of MDTs is linked to a higher probability of patients receiving active treatment [17]. Thus, MDT‐led decision making might expand the pool of patients who may benefit from standard of care (SoC) curative therapy and potentially improve recruitment to clinical trials.

However, the benefits of MDT‐led decision making must be weighed against the significant associated cost (£415 [$550 or €486] for every new patient discussed), particularly for cases where interspecialty input is not required and decision making can be performed per‐protocol [18].

In this review, we highlight the value of a multidisciplinary approach to diagnosis, staging, treatment decision making, and adverse event management in NSCLC.

## MDTs: Impact and Benefit of Joint Decision Making

A wider and more standardized implementation of MDTs could help to address a number of decision‐based factors that can influence patient outcomes, including differences in local guidelines and procedures or the type of treating center and referral route.

The use of MDTs is considered in several national and international guidelines that are designed to ensure rapid diagnosis, staging, and decision making and the provision of radical treatment whenever possible. For example, the National Optimal Lung Cancer Pathway developed by the U.K.'s National Health Service requires a case be discussed by the MDT within 3 weeks of initial triage and any additional investigations performed within the following week [19]. Similarly, the Dutch Association of Physicians for Pulmonary Diseases and Tuberculosis recommend a multidisciplinary approach to reduce diagnostic and therapeutic delays, with targets stipulating that 80% of patients should have a complete diagnosis within 3 weeks of presentation; of these, 80% should start treatment within 2 weeks of diagnosis [20]. Furthermore, regional guidelines in the U.S. (National Comprehensive Cancer Network), Europe (European Society for Medical Oncology [ESMO]), and Asia (ESMO guidelines adapted and endorsed by a collective of six Asian countries as the Pan‐Asian Guidelines Adaptation [PAGA]) stress the particular importance of MDT collaborative decision making when a biopsy (and therefore histological confirmation) is impossible during diagnostic evaluation and in disease settings with multiple treatment options (e.g., borderline resectable disease, synchronous oligometastatic disease), which require case‐by‐case evaluation [3, 21, 22, 23, 24].

Decision making, and thus patient outcomes, can also vary according to the type of treatment center providing patient care. A retrospective study of the U.S. National Cancer Database identified a clear disparity in the survival of patients with NSCLC between academic and community centers, a gap that widened significantly over time [25]. The investigators reasoned that academic centers improved their adaptability to the introduction of novel treatment methods (e.g., tyrosine kinase inhibitors) dependent on molecular analysis or histology, which contributed to the widening of this gap in patient survival. Academic centers likely have earlier experience of novel treatments through participation in clinical trials. It is also likely that academic centers have a greater variety of specialists and thus may be better equipped to implement effective MDT‐led decision making, especially for patients with borderline radically treatable disease, a previously observed phenomenon [26]. The U.K. National Lung Cancer Audit identified that, even after adjustment for case‐mix factors, patients were more likely to undergo surgical resection if first seen with an MDT based in thoracic surgical centers, probably because of surgical peer review of decision making [26, 27]. However, one must also consider other factors that contribute to differences in outcomes between academic and community centers, including patient selection (e.g., better performing patients are able to travel to academic centers), better and faster diagnostics [28], and potential enrollment into clinical trials.

Regardless of where patients ultimately receive their treatment, initial referral route can lead to treatment decision disparities. Approximately 50% of patients with lung cancer in the U.K. were referred by their general practitioner (GP), whereas 32% of patients were diagnosed following emergency presentation [29]. Those referred by GPs were approximately five times more likely to undergo tumor resection (with or without adjuvant and/or neoadjuvant therapy) than patients with emergency presentation [30]. Indeed, emergency patients tend to be “more unwell” with poorer performance status, resulting in increased mortality rates compared with electively referred patients [31, 32]; this suggests referral route alone is unlikely to account for differences in treatment decisions. However, such inconsistencies could be reduced through standardized application of an MDT at the point where a patient enters care for their suspected NSCLC, regardless of referral route. Additionally, MDT case review can serve to highlight patients suitable for clinical trials, enabling systematic screening and tracking of new patients and maximizing recruitment.

Regional bodies have also endeavored to address this variability in decision making. In 2013, a European Expert Group convened to develop a “best practice” guide for diagnosis. The key outcome was a need for standardized procedures (e.g., biopsy size and quality, pathologist involvement, molecular testing and reporting of test results, and administrative procedures) [33]. Similar statements and recommendations from the British Thoracic Society and the European Respiratory Society further support the role of a multidisciplinary approach to patient care [10, 34]. In the following section, we aim to build and expand on these frameworks by discussing the major considerations for an MDT in the treatment of patients with NSCLC, based on stage of disease.

## MDT Treatment Choices

Recommended treatments (per guidelines) at each stage of NSCLC are summarized in Table 1. We discuss these recommendations focusing on areas of controversy requiring MDT discussion and consensus, as well as possible additional specialist MDT input, below. At every stage, individual patient decisions should be considered and the MDT should endeavor to identify patients’ suitability for clinical trial enrollment [35].

**Table 1:** Treatment guideline recommendations by tumor stage and areas of controversy for MDT discussion

NSCLC tumor stage	Usual treatment recommendation	Areas for discussion in which MDT review may benefit	Areas in which additional MDTs may input into care
I	Surgery, ideally lobectomy by VATS SBRT for medically comorbid patients	SBRT as either an alternative or preferred option Role of catheter ablation vs. SBRT, especially if comorbidities such as fibrotic lung disease Enrollment into clinical trials Management of endolumenal disease Consideration of patient's wishes	Input from regional advanced radiotherapy MDT Input from specialist pulmonary nodule management MDT Input from regional specialist MDT
II	Surgery, ideally lobectomy by VATS	Accurate mediastinal staging SBRT as an alternative or preferred option if node negative Role of catheter ablation vs. SBRT, especially if comorbidities such as fibrotic lung disease Review of resection quality and decision making on role of adjuvant chemotherapy Staging, diagnosis and treatment of medically comorbid patients, especially those with poor lung reserve Enrollment into clinical trials Consideration of patient's wishes	Input from regional advanced radiotherapy MDT Input from specialist pulmonary nodule management MDT
III	Multimodality therapy with/without surgery, radiotherapy, or durvalumab	Accurate mediastinal staging Agreed multimodality approach Diagnostic specimen PD‐L1 status Management of borderline resectable patients Staging, diagnosis, and treatment of medically comorbid patients, especially those with poor lung reserve Systemic therapy; e.g., immunotherapy adverse event management Enrollment into clinical trials Consideration of patient's wishes	Input from regional multimodality therapy MDT
IV	Systemic therapy	Pathological sampling to maximize yield for biomarker evaluation Management of synchronous or metachronous oligometastastic disease Management of oligoconsolidation or oligoprogression Evaluation of sites to rebiopsy on acquired resistance to systemic therapy Systemic therapy e.g. immunotherapy adverse event management Pleural effusion management Enrollment into clinical trials Consideration of patient's wishes	Input from GTAB to advise systemic therapy Input from regional neuroscience MDT on management of untreated or treated (reactive) CNS metastases Input from supra‐regional MDT to advise on treatments for patients with rare genomic variants Immunotoxicity MDT to advise on toxicity identification and management Input from regional advanced radiotherapy MDT

Abbreviations: CNS, central nervous system; GTAB, genomic tumor advisory board; NSCLC, non‐small cell lung cancer; MDT, multidisciplinary team; PD‐L1, programmed cell death ligand‐1; SBRT, stereotactic body radiotherapy; VATS, video‐assisted thoracoscopic surgery.

### Stage I NSCLC: Surgery Versus SBRT

Stage I disease is ideally treated with surgical resection, preferentially video‐assisted thoracoscopic surgery lobectomy; the best outcomes are associated with an R0 resection [4, 36, 37, 38, 39]. Despite this, surgery rates have fallen in Europe and the U.S. in recent years (but not in England and Wales) [40, 41]. Where MDTs are currently employed in surgical decision making, two possible explanations for this decline arise: an MDT might conclude that surgery is clinically inappropriate based on patient characteristics (such as comorbidities) and recommend other treatments [15]; alternatively, patients who are borderline unfit or unwilling to undergo surgery may choose a less invasive treatment option.

Stereotactic body RTx (SBRT) may be considered in patients unfit for, or who decline, surgery and has been associated with increased overall survival (OS) for RTx and surgical patients alike [40]. Thus, the suitability of individual patients for SBRT should be discussed among members of the MDT and particularly with a thoracic surgeon [42]. Moreover, a specialist advanced radiotherapy MDT may be consulted for complex situations (e.g., very mobile tumors in proximity to the left hemidiaphragm or the heart, or when coexistent interstitial lung disease is present), where a catheter‐based ablative approach may be more appropriate. Shared decision making with patients should be performed in all cases to ensure the patient understands the risks, side effects, and alternative treatments available [43]. In addition, the role of pathological mediastinal/hilar staging may warrant MDT discussion contingent on positron emission tomography–computed tomography (CT) findings to exclude occult involvement [44]. Finally, in a real‐world analysis, only 54% of patients with stage I disease underwent resection and 24% received no active treatment, representing a major opportunity for MDT decision making to improve patient survival [45]. Many patients with stage I NSCLC have background pulmonary nodules, with management requiring rigorous systematic evaluation [46]. Many countries have reorganized MDT functioning to develop a specialist pulmonary nodule MDT, where imaging can be reviewed according to guidelines with dedicated radiologists to allow quantification.

### Stage II NSCLC: Surgery Versus SBRT Versus Chemoradiotherapy

Similar to stage I, where surgery with lobectomy remains the gold standard where possible, the possibility of lymph node involvement necessitates that increased importance is given to preoperative mediastinal staging [47, 48, 49]. This is especially relevant in patients with borderline fitness for a radical approach, and, for patients undergoing surgery, review of the postoperative pathology report is valuable. MDT review is important in confirming the “R” status of the tumor [35], establishing that a complete resection has been performed [50], and to review the pathological nodal stations (harvested and involved) alongside margins.

Therefore, MDT review of resection cases, with pathology reports and preoperative staging alongside surgical review, remains the cornerstone to confirming complete resection and decision making regarding appropriate adjuvant therapy, allowing adequate discussion on patient selection for adjuvant CTx [23, 47].

A number of trials are also evaluating the potential role of (neo)adjuvant immunotherapy in early‐stage NSCLC, with MDT discussion critical to ensuring eligible patients are enrolled [35].

### Stage III NSCLC: Multimodal Therapy of Chemoradiotherapy and Surgery Versus Chemoradiotherapy with Durvalumab

Stage III NSCLC is a highly heterogeneous disease classification treated with curative‐intent multimodality therapy [36, 51]. However, as almost equal heterogeneity exists in decision making around diagnostic, staging, and therapeutic choices [3, 4, 52], close MDT working is essential. Again, MDT consensus around mediastinal staging is essential to planning the optimal approach [49, 53], particularly regarding operability and establishing RTx target volumes [54].

A tumor should be deemed unresectable based on evaluation within an MDT that includes an experienced thoracic surgeon [24]. For cases of borderline resectable tumors, MDT discussion involving the relevant specialists (including at least one surgeon) is vital in the optimal management of patients as there is no defined “best approach”; this is reflected in recommendations across multiple national and international guidelines [3, 21, 24]. The increasing prevalence of multimodality treatment for stage III NSCLC emphasizes the need for continuous and regular feedback between MDT members to ensure guideline compliance and the local audit of outcomes.

Based on results from the PACIFIC trial, which showed that treatment with durvalumab after concurrent CTx and RTx significantly increased progression‐free survival (stratified hazard ratio [HR], 0.52; 95% confidence interval [CI], 0.42–0.65; *p* < .0001; median, 16.8 vs. 5.6 months) and OS (stratified HR, 0.68; 99.73% CI, 0.47–0.997; *p* = .0025; median not reached vs. 28.7 months) in patients with stage III, unresectable NSCLC [55, 56, 57, 58, 59, 60, 61], durvalumab following chemoradiotherapy (CRTx) was rapidly established as the SoC for this population. However, the European approval of durvalumab for unresectable stage III NSCLC limits use to tumors with programmed cell death ligand‐1 (PD‐L1) expression on ≥ 1% of tumor cells [7]. This stipulation was based on an exploratory subgroup analysis in PACIFIC requested by the European Medicines Agency, where evaluable PD‐L1 expression data were only available for 66% of patients [60]. This caveat makes accurate testing for PD‐L1 expression in patients with stage III disease particularly crucial in European centers; however, testing‐related difficulties may emerge, requiring MDT assessment of the stage III patient treatment paradigm. Tissue samples retrieved must have adequate tumor content (quality and quantity) to permit a full subtype diagnosis and genotyping as appropriate, as well as PD‐L1 immunohistochemical testing. Tissue availability will, in turn, depend on MDT discussion to ensure appropriate sampling while respecting patient safety and need for diagnosis, as post‐CRTx biopsy sampling is not feasible.

Tumor samples are used for both core diagnosis and biomarker testing, and the MDT typically needs rapid results. Therefore, the choice of which tests to order should be made based on local expertise and protocols, need, cost, and tissue availability [62, 63]. To enable accurate assessment of potential benefit, PD‐L1 expression results should be communicated as a percentage of evaluable tumor cells expressing PD‐L1, rather than simply “high” or “low” [7]. Given that the majority of patients with stage III NSCLC undergo endobronchial ultrasound mediastinal staging, it is imperative that these samples are deemed locally suitable for PD‐L1 testing, as previously validated in the BLUEPRINT2 study, to avoid patients undergoing an additional biopsy for predictive biomarker testing [64].

In addition to ongoing trials, there is emerging evidence to suggest that the use of immunotherapy in a neoadjuvant capacity prior to surgery could potentially improve outcomes [65]. In an exploratory trial of patients with stage IIIA resectable NSCLC treated with platinum‐based CTx plus nivolumab before undergoing surgery (*n* = 13), the overall response rate was approximately 85%, with 9 patients (69.2%) achieving complete pathological response (95% CI, 38.6–90.9%) [66]. The SAKK 16/14 trial demonstrated a 1‐year event‐free survival rate of 73.3% with CTx and durvalumab, followed by surgery and 12 months of postoperative durvalumab, in patients with resectable stage IIIA NSCLC [67]. The TOP 1501 trial features patients with stage IB, II, or IIIA NSCLC being treated with neoadjuvant pembrolizumab monotherapy, followed by postoperative CTx and pembrolizumab [7]. Thus, in this highly heterogeneous disease group, clinical trial enrollment is critical for progression toward greater treatment consensus.

Additional MDT review may be required for patients with stage III NSCLC, particularly around surgical and RTx assessments and especially the delivery of trimodality therapy (CTx‐RTx‐surgery), where MDT and center expertise is consistently identified as a critical factor in decision making. Hence, such patients may need to be discussed at a regional specialist MDT with expertise in such complex therapy, particularly for borderline patients, such as those requiring large radiation volumes.

Patient preference is a key part of any treatment decision making process in stage III NSCLC: patients may decline surgery, fail to adhere to treatment schedules, or be intolerant of treatment‐adverse events. Patient feedback about their experiences with MDTs has highlighted a desire for transparency. If resection is not an option, then an explanation of why that is the case may make patients more likely to adhere to the alternative [68]. Likewise, if a multimodality treatment regimen is recommended it is prudent for the entire treatment plan to be explained as early as possible to prepare patients and carers.

### Stage IV NSCLC: Role of Local Treatment and Pathological Oversight

Stage IV disease is incurable for the vast majority of patients. Thus, whether any additional survival benefit can be gained in a systemically disseminated disease by optimally treating local sites is hotly debated. ESMO guidelines suggest that the use of local ablative therapies should be based on the extent of tumor metastasis [23]; there is limited evidence that patients with synchronous oligometastatic disease (featuring only 1–5 metastases in up to 3 organs) may experience long‐term disease‐free survival following systemic therapy and SBRT or surgery [69]. It is highly recommended that these cases should be discussed extensively within the MDT because evidence for local consolidation is inconclusive and clinical decision making will ultimately depend on specific patient characteristics. Whether for synchronous/metachronous oligometastatic disease or oligoprogressive disease on long‐term systemic therapy, ESMO/PAGA treatment guidelines emphasize the importance of identifying such patients through MDT discussion and enrolling them in clinical trials (to establish an evidence base for the use of local ablative therapies in this disease setting) [22, 23]. Notably, results from a recent phase II trial demonstrated OS benefit with local radical consolidative therapy (definitive RTx and/or surgical intervention) versus maintenance treatment and observation (41.2 months vs. 17.0 months, respectively: HR, 0.46; 95% CI, 0.21–0.99; *p* = .048) [70]. However, as the sample size was relatively small (25 vs. 24 patients in the local consolidative therapy vs. maintenance and observation arms, respectively) and the degree of benefit derived appeared to vary according to disease variables (number of metastases, mutation status), further investigation is warranted in this area to definitively adjudicate the optimal treatment approach. MDT discussion regarding these patients should therefore heavily focus on the assessment of patient suitability for ablative therapies and, ideally, clinical trial enrollment.

Although genotyping to identify driver gene alterations and PD‐L1 testing of tumors are now standard in stage IV NSCLC, performing genotyping procedures is often challenging [71]. The emergence of multiple molecularly targeted therapies for advanced NSCLC over the past 15 years reinforces the critical need for a pathologist (and potentially a molecular biologist, as required) on the MDT to facilitate optimal sample‐handling for successful and informative molecular analyses, minimize tissue wastage and optimize sample flows (as molecular laboratories may not be colocated with diagnostic centers or pathology laboratories), and minimize delays to result publication. Moreover, given that the current World Health Organization classification of thoracic malignancies now includes recommendations on small biopsies, the presence of a pathologist is critical in ensuring the appropriate histological subtype has been identified to aid prior predictive utility of genotyping [72]. As genotyping technologies progress, the identification of rare and indeterminate genetic variants is increasing, particularly in patients with oncogene‐addicted disease whose tumors may undergo genotyping at multiple stages during acquired systemic therapy resistance. Here, MDT decision making can be critical, not only to identifying lesions suitable for rebiopsy to identify drug resistance mechanisms but also via genomic tumor advisory boards (GTABs) that can review the data underpinning a genetic variant and give appropriate advice on actionability. Finally, for patients with central nervous system (CNS) involvement, the optimal treatment strategy will require discussion with neuroscience specialists, alongside those from pathology or molecular diagnostics, to best decide the role of stereotatic radiosurgery upfront versus on demand at relapse, with optimal systemic therapy using a CNS‐penetrant drug.

## The Role of the MDT in Adverse Event Management

Adverse events (AEs) are an unavoidable consequence of all known cancer treatments [3]. Specialist lung cancer nurses and palliative care specialists play a crucial role in monitoring AEs, coordinating the involvement of physicians outside of the core MDT, and (together with nutritionists) facilitating optimal preparation of patients for, and support during, treatment [11, 73, 74].

CTx is associated with side effects that can seriously affect quality of life (and potentially decrease adherence), ranging from anemia, nausea and vomiting to hemoptysis, and severe neutropenia [75, 76]. One AE associated with CRTx (and immunotherapy) that is of particular interest for the MDT is pneumonitis [77]. Considering the differential diagnoses of infection, progression, immune‐related‐, or postradical radiation‐induced pneumonitis, MDT input, from radiologists, radiation oncologists, pulmonologists, and medical oncologists, can be instrumental in confirming the diagnosis through image interpretation and review of radiation treatment volumes. Given a need for early intervention, additional investigations, such as an early investigative bronchoscopy (with differential cell count on lavage) to exclude an immune‐related pneumonitis or cardiology involvement to exclude cardiac failure or myocarditis, may be indicated [78, 79]. Similarly, a surprisingly high rate of pneumocystis pneumonia occurs in patients after radical radiotherapy or while on long‐term steroids [80] for other immune‐related adverse events, presenting with a subacute pneumonitis picture; in this situation, MDT review can be crucial to ensuring optimal management.

As immune checkpoint inhibitors are implemented into wider routine practice, specialist MDTs can be considered to review immune‐mediated AEs alongside potential drug benefits (immunotoxicity MDTs). As indications broaden and combination immunomodulatory therapies are delivered, this may be particularly important in regions with initially limited immune checkpoint inhibitor experience. Immunotoxicity MDTs would aim to share knowledge regarding service delivery, AE management, and patient selection, alongside mortality review, and ensure robust organizational phamacovigilance. Such immunotoxicity MDTs may be composed of oncologists from different tumor types and with expertise in both systemic therapy and RTx (alongside other relevant specialists, e.g., pulmonary physicians, endocrinologists, gastroenterologists, critical care physicians, pharmacists, nurses) and may become increasingly important as novel methods of immunomodulation (e.g. cell therapies) are more widely implemented.

## MDT Organization and Role

Although it is now clearly recognized that MDT working is a key quality measure [8], the associated time spent and financial cost may be limiting. Different solutions for MDT working may be considered to streamline this process, contingent on the health care system. For example, patients who fit into a predefined MDT staging and treatment pathway (e.g., staging, diagnosis, and surgery for stage I–II NSCLC without comorbidity) may be efficiently managed by a local MDT, whereas patients with borderline indications (e.g., stage I NSCLC with poor lung function or comorbidities) or requiring MDT discussion (e.g., radically treatable stage III NSCLC) may be considered for a supra‐regional MDT, where there may be additional experience afforded by the cumulative volume of complex cases. As CT screening becomes more widely implemented, a dedicated MDT should discuss management of detected nodules above certain sizes [8]. Supra‐regional or even national MDTs could be considered for rare defined conditions, to leverage a greater volume of experience. For example, the French nationwide RYTHMIC network, dedicated to the management of thymic epithelial tumors, ensures coverage by 14 regional expert centers, aiming to disseminate the highest standards for diagnosis and treatment and promote collaborative research [81]. The twice‐monthly virtual RYTHMIC MDT meetings feature a minimum of three expert teams discussing the management of patients according to network guidelines, with patient details prospectively collected in a database. Similarly in the U.K., with the implementation of whole genome sequencing, supra‐regional GTABs review somatic and germline patient data and directly feedback to local MDTs or treating physicians [82]. Although traditionally recognized as important for patients with metastatic NSCLC, as indications broaden and sequencing technologies identify rare variants with increasing frequency, GTABs may become increasingly important for radically treated NSCLC. Finally, an additional MDT of importance for lung cancer patients would be a neuroscience MDT, as implemented at a regional level in England. Here, patient cases with CNS involvement can be evaluated to determine metastatic involvement (or potential for radionecrosis in treated patients) and decide an appropriate treatment plan. Moreover, as the complexity of treatment decision making increases, we also recognize the role of information technology–based assistance; for example, GTABs employ such tools to identify trials suitable for patient genotype, a concept that can be broadly expanded for rare diseases or supra‐regional MDTs. As diagnostic and treatment indications continue to increase at a pace for thoracic malignancies, in parallel to regional changes in health care service delivery, there will inevitably be ongoing change to each nation's requirement for local or supra‐regional MDT working. The limited availability of clinician time and financial resources, and the relative scarcity and consequent value of academic clinicians, will remain major obstacles to wider implementation.

## Conclusion

With the treatment landscape in NSCLC continuously evolving because of the integration of new treatments such as SBRT and immune checkpoint inhibitors into routine care, the broad expertise of an MDT is required to design the most effective treatment plan and thereby attain maximal clinical benefit. The effectiveness of MDTs in improving patient outcomes requires further study.

## Author Contributions

**Conception/design:** Sanjay Popat, Neal Navani, Keith M. Kerr, Egbert F. Smit, Timothy J.P. Batchelor, Paul Van Schil, Suresh Senan, Fiona McDonald

**Manuscript writing:** Sanjay Popat, Neal Navani, Keith M. Kerr, Egbert F. Smit, Timothy J.P. Batchelor, Paul Van Schil, Suresh Senan, Fiona McDonald

**Final approval of manuscript:** Sanjay Popat, Neal Navani, Keith M. Kerr, Egbert F. Smit, Timothy J.P. Batchelor, Paul Van Schil, Suresh Senan, Fiona McDonald

## Disclosures

**Sanjay Popat:** Bristol Myers Squibb, Roche, Takeda, AstraZeneca, Pfizer, Merck Sharpe & Dohme, EMD Serono, Guardant Health, Abbvie, Boehringer Ingelheim, OncLive, Medscape (Other‐Personal fees); **Neal Navani:** Hitachi, Pentax, Cook and AstraZeneca (Other‐Educational grants, Other‐Fees‐institution); **Keith M. Kerr:** Abbvie, Archer Diagnostics, AstraZeneca, Bayer, Boehringer Ingelheim, Celgene, Eli Lilly & Co, Merck Serono, Merck Sharpe & Dohme, Novartis, Pfizer, Roche, Ventana, Medscape, PER, Prime Oncology, Springer (Other‐Fees for consultancy and/or lecturing); **Egbert F. Smit:** AstraZeneca (Advisory board); **Timothy J.P. Batchelor:** Medtronic, Johnson & Johnson, AstraZeneca (Other‐Fees); **Paul Van Schil:** National Cancer Institute external expert (France), AstraZeneca, Merck Sharpe & Dohme (Institutional fees); **Suresh Senan:** AstraZeneca, Merck, Celgene, Eli Lilly & Co (C/A), AstraZeneca, ViewRay Inc., Varian (RF); **Fiona McDonald:** Accuray, AstraZeneca (C/A), AstraZeneca, Elekta (Other‐Fees).

(C/A) Consulting/advisory relationship; (RF) Research funding; (E) Employment; (ET) Expert testimony; (H) Honoraria received; (OI) Ownership interests; (IP) Intellectual property rights/inventor/patent holder; (SAB) Scientific advisory board
